# Wear Behaviors of Carbon–Chromium Carbide–Chromium Multilayer Coatings Prepared by Reactive High-Power Impulse Magnetron Sputtering

**DOI:** 10.3390/ma14247694

**Published:** 2021-12-13

**Authors:** Chin-Chiuan Kuo

**Affiliations:** Department of Mechanical and Computer-Aided Engineering, National Formosa University, Huwei, Yunlin County 63201, Taiwan; cckuo@nfu.edu.tw; Tel.: +886-5631-3492

**Keywords:** chromium carbide, carbon, ethyne, high-power impulse magnetron sputtering, wear

## Abstract

Carbon–chromium carbide–chromium multilayer coatings were deposited by utilizing reactive high-power impulse magnetron sputtering with alternating various ratios of ethyne and argon mixtures under a constant total deposition pressure, target pulse frequency, pulse duty cycle, average chromium target power, and total deposition time. Two different alternating gas mixture periods were applied to obtain films with different numbers of layers and lamination thicknesses. The results show that the reduction in the modulation period effectively affects the elastic modulus and the subsequent ratio of hardness to elastic modulus (H/E) of the whole coating, which helps adapt the elastic strain in the coating. This improves the adhesion strength and wear resistance of coatings at room temperature. However, with the increase in wear test temperature, the difference between the wear behaviors of two types of coatings becomes inconspicuous. Both types of coatings lose the wear resistance due to the decomposition of hydrocarbon and the oxidation of the chromium content in the films.

## 1. Introduction

By controlling the process parameters, various combinations of carbon and chromium, including chromium-based alloys, chromium carbides of different stoichiometric compositions, carbon-based materials and multiphase composites can be synthesized by utilizing plasma-enhanced physical vapor deposition techniques [1,2,3,4,5,6,7,8,9,10,11,12,13,14,15,16,17,18,19,20]. Each compound or phase exhibits individual mechanical, chemical properties, and accompanying application performances. The chromium content usually offers great oxidation and corrosion resistance due to the formation of a passive chromium oxide layer, which blocks active substances [21]. The amorphous carbon phases exhibit a low coefficient of friction and a higher ratio of hardness to elastic modulus, which usually provides a superior wear resistance [6,17,22]. The chromium carbides show high hardness and chemical stability at high temperature [22]. Based on these characteristics, chromium carbide coatings have been commonly used as wear and corrosion-resistant protective coatings [3,6,17,22]. Due to their wide adjustable range of mechanical properties, they can also be used as the adhesion enhancement interlayer between metallic substrate and carbon-based topcoat [18,22].

In our previous works [19,20], Cr–C coatings with various fractions of chromium, chromium carbide, and amorphous hydrogenated carbon (a-C:H) phases can be synthesized by using reactive high-power impulse magnetron sputtering (HiPIMS) with the ethyne flow ratio adjustment. It was found that the microstructure of the obtained Cr–C film changes from a loose staking-clusters structure into a columnar structure, then into a dense glassy structure, and finally into a crystalline columnar structure with an increasing chromium concentration. The main phases of the coating which consists of Cr and carbide are glassy brittle due to the high hardness, high elastic modulus and the tendency to be amorphous. The coating containing more amorphous hydrogenated carbon exhibits a relatively lower hardness and elastic modulus. This difference in the mechanical properties affects both the adhesion and service life of the coatings under wear environments [23]. Although it is believed that the HiPIMS enables the high-degree ionization of the sputtered species and the feasibility of tailoring a structure by applying substrate biasing, this feature seems to be unable to overcome the glass-forming nature of Cr–C compounds at a relative lower deposition temperature [24,25,26,27,28].

To improve the wear resistance of hard coatings, Leylands et al. proposed a nanocomposite multilayer structure which consists of the alternately laminated hard ceramic layers and ductile metallic layers [29,30]. This design effectively raises the ratio of hardness to elastic modulus and improves the toughness of the coating to suppress the fatigue crack propagation in the coating [31]. Decreasing the thickness of each layer in such a periodic multilayer also reduces the compressive stress in the coating [32,33]. This kind of multilayer structure has also been attempted in soft-carbon-fiber-reinforced epoxy composites substrate in order to resist bending stress, and a low-friction amorphous diamond-like carbon toplayer has effectively improved the overall wear resistance of the multilayer coating [34]. However, there is also an optimal range of total coating thickness of machining tools for particular cutting conditions [35]. 

Based on the above research reports and the previous experiences, multilayered carbon–chromium carbide–chromium composite coatings were deposited by reactive high-power impulse magnetron sputtering, utilizing the chromium target and sequenced alternating argon/ethyne gas flow ratio in this study. Two kinds of multilayered coating architectures were deposited. Those coating architectures have the same fraction ratio of carbon, chromium, and chromium carbide, but have different single layer thicknesses. The mechanical properties and wear behaviors under various temperature of the two coatings were compared.

## 2. Materials and Methods

Cr–C multilayered coatings were deposited onto polished AISI M35 high-speed steel disks with a dimension of 55 (*Φ*) mm × 10 (*h*) mm in an HiPIMS deposition system as described in previous works [19,20]. A rectangle chromium target (432 (*l*) × 76 (*w*) × 13 (*h*) mm) with an unbalanced magnetron was connected to a unipolar HiPIMS power supply (TRUMPF, Hüttinger TruPlasma Unipolar 4001). The M35 disks were placed facing the chromium target at a distance of 100 mm, and the substrate holder was connected to the unipolar substrate bias power supply (TRUMPF Hüttinger TruPlasma Bias 4010 G2).

After vacuuming the deposition chamber, the argon glow discharge plasma substrate cleaning proceeded in a 2.7 Pa argon atmosphere by applying direct current −1000 V substrate bias voltage for 60 min. After argon plasma cleaning, the HiPIMS pulse parameters were set to the pulse width of 60 μs, pulse frequency of 175 Hz, and average target power of 1.5 kW. The HiPIMS discharge plasma was initiated on the Cr target in 0.8 Pa argon atmosphere, and the −1000 V substrate bias voltage was switched to the unipolar pulsed mode with a pulse width of 105 μs synchronized with the target HiPIMS pulses. Such high substrate bias voltage was applied to achieve the Cr ion bombardment in order to enhance the adhesion of coating. A thin Cr layer (approximately 80 nm thick) was deposited after 2 min Cr ion bombardment. Using the synchronized pulsed substrate bias can prevent the drop of substrate voltage and facilitate the process of selectively attracting ions and the charged species waves hitting the substrate [19,20,36,37]. During the argon plasma cleaning and the Cr ion bombardment, the substrate temperature was moderately raised to approximately 200 °C. After Cr ion bombardment, the synchronized substrate bias voltage was mitigated to −100 V. The C_2_H_2_/Ar gas flow ratio was sequentially changed to 1:14 to deposit a hydrogenated carbon layer, the C_2_H_2_/Ar gas flow ratio 1:24 to deposit the chromium carbide layer, and only Ar gas to deposit the chromium layer, then the C_2_H_2_/Ar gas flow ratio 1:24 to deposit chromium carbide layer. The ratio of deposition time of the hydrogenated carbon, chromium carbide, chromium, and chromium carbide layers were 3:1:2:1. The obtained thickness ratio of these four layers was approximately 23:7:11:7. This gas flow sequence was arranged based on the results of a previous work [20] and to moderate the changes of target voltage and current. The chromium carbide phase exhibited highest hardness, followed by the hydrogenated carbon phase, and the chromium phase. The ranking of the ratio of hardness to elastic modulus (H/E) of each phase was different. The hydrogenated carbon phase had highest H/E value, followed by the chromium carbide phase, and the chromium phase. In this deposition sequence, the flow ratio of C_2_H_2_ for depositing each layer increased and reduced step by step to moderate the changes of target voltage and current between the metallic mode and compound mode depositions [20]. After periodically depositing this quad-layer one or four times, a hydrogenated carbon (a-C:H) top layer (approximately 360 nm thick) was deposited. During the deposition, both the HiPIMS pulse voltage and current varied with the C_2_H_2_ gas flow ratio to keep a constant pulse width, pulse frequency, and average target power of 1.5 kW. The variation range of the pulse voltage and pulse current was referred to in a previous work [20]. The deposition time was 32 min and the total thickness of the coating was approximately 2.8 μm. Two coating architectures were developed. One consisted of only a single period of quad-layer (deposition time 12, 4, 8, 4), labeled as 1T, and the other one consisted of four periods of quad-layer (deposition time 3, 1, 2, 1), labeled as 4T. The volume fractions of each phase in these two types of coatings were the same.

The thickness of each layer was estimated based on the results of previous works [19,20,36] and the total coating thicknesses were measured using a Calotest Compact Calotester (CAT^2^c, Anton Paar, Graz, Austria). The adhesion strength of the coatings was evaluated by the indents of HR-400 Rockwell hardness indentation machine (Mitutoyo, Tokyo, Japan), as described in the VDI (Verein Deutscher Ingenieure, Association of German Engineers) guideline 3198. The hardness and the modulus of elasticity of the Cr–C films were evaluated by applying nanoindentation (TTX-NH3, Anton Paar, Graz, Austria). The indentations proceeded by applying a maximum load of 40 mN with a 10 s pause. The mean hardness of one sample was averaged from 12 indents.

The wear tests of the coated samples were conducted using a ball-on-disc abrasive high-temperature tribometer (THT, Anton Paar, Graz, Austria) in accordance with the standard ASTM G 99-17 [38] at various temperatures of 25 °C, 150 °C, 300 °C and 450 °C. Al_2_O_3_ (*Φ* 6 mm) balls were used as the counterpart, and sliding on a track of 20 mm diameter at all test temperature. The normal load was 7 N, and the rotation speed of coated disc was 400 rpm. A Raman spectrometer (PROTRUSTECH, Tainan, Taiwan) was used to detect the presence of carbon phases in the toplayer and oxides formed at high temperature.

## 3. Results and Discussion

### 3.1. The Structure and Mechanical Properties of Coatings

**Figure 1:**
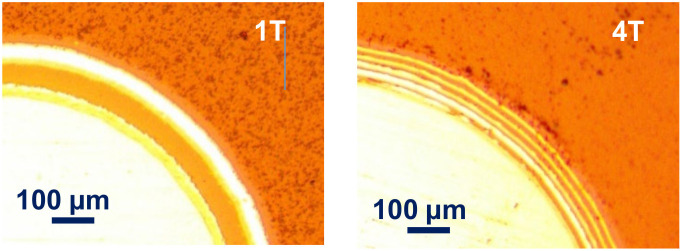
Optical microscope images of the craters of Calotest on the Cr–C multilayer coatings with 1 quad-layer (**1T**) and 4 quad-layer (**4T**) deposited on the AISI M35 substrate with a substrate bias of −100 V at 0.8 Pa.

**Figure 2:**
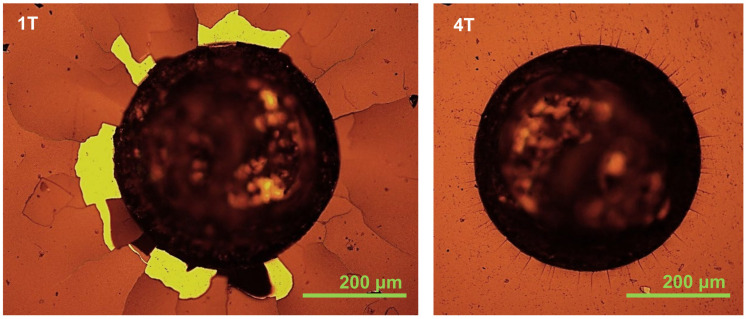
Optical microscope images of Rockwell indents on the Cr–C multilayer coatings with 1 quad-layer (**1T**) and 4 quad-layer (**4T**) deposited on the AISI M35 substrate with a substrate bias of −100 V at 0.8 Pa.

From the images of the craters of the Calotest shown in Figure 1, the coating architectures are in line with the expected design. The sample 1T shows a 6-layer architecture (Cr bond coat + 1 period of quad-layer + a-C:H top coat), and the sample 4T presents an 18-layer architecture (Cr bond coat + 4 periods of quad-layer + a-C:H top coat). The Rockwell indents of the two coatings are shown in Figure 2. According to the VDI 3198 evaluation, the sample 4T presents an excellent adhesion strength quality HF1, but the sample 1T shows a poor adhesion strength quality HF5. This indicates that the reduction in the single layer thickness (modulation period) does improve the tolerance to the strain between the substrate and the multilayer coating.

**Figure 3:**
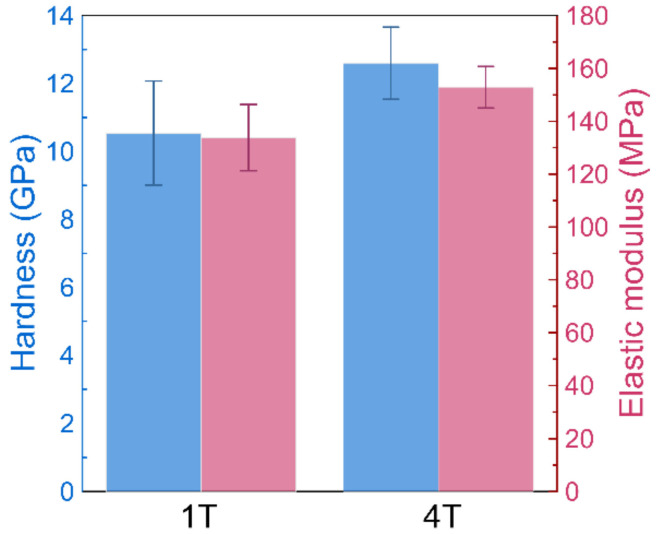
Mean values and standard deviations for nanoindentation hardness (blue bars) and elastic modulus (red bars) for Cr–C multilayer coatings with 1 quad-layer (**1T**) and 4 quad-layers (**4T**) deposited on the AISI M35 substrate with a substrate bias of −100 V at 0.8 Pa.

The hardness and elastic modulus of the two coatings measured by the nanoindentation are shown in Figure 3. The sample 4T with a smaller modulation period (thinner single layer and more layers) exhibits a higher hardness, a higher elastic modulus, as well as a larger ratio of hardness to elastic modulus (H/E). The better adhesion, the higher hardness, and the larger H/E value imply the superior wear resistance of sample 4T. 

### 3.2. Wear Behaviors of Coated Samples at Different Test Temperatures

**Figure 4:**
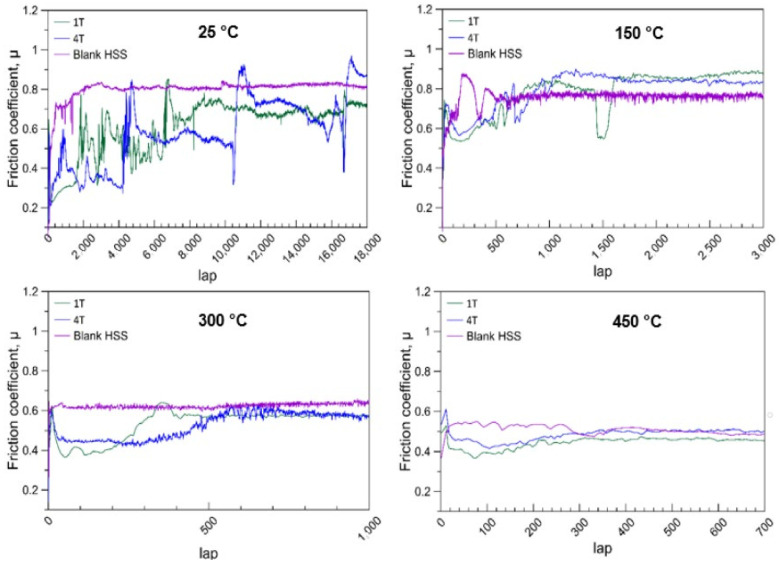
Friction coefficients depending on the lap number (cycles) for the tribological contact of an alumina ball (6 mm) in ball-on-disk testing with uncoated and two different multilayer-coated AISI M35 under a 7 N load at 25 °C, 150 °C, 300 °C, and 450 °C.

Figure 4 shows the evolution of the friction coefficients in contact with Al_2_O_3_ counterpart balls in function of the contact lap number obtained at different wear test temperatures. At 25 °C, the coatings reduced the friction coefficients in the beginning. The friction coefficients of both coated samples increased with the lap number. The friction coefficients of sample 1T reached a steady value which was near the steady value of blank M35 after 8400 laps, and that of sample 4T occurred after 17,000 laps. This shows that the coating with a smaller modulation period (thinner single layer and more layers) does provide a superior wear resistance. However, with the increasing wear test temperature, the failure lap number of coating decreases tremendously. In addition, the difference between sample 1T and 4T becomes inconspicuous. At 450 °C, both coatings did not show any wear resistant function. These results indicate that the thermal stability of this multilayer might be not sufficient. This trend was also reported by Gassner et al. [12]. They inferred that the a-C:H phase decomposed and transformed into a metastable fcc Cr–C phase due to the loss of bonded hydrogen at temperatures above 200 °C.

**Figure 5:**
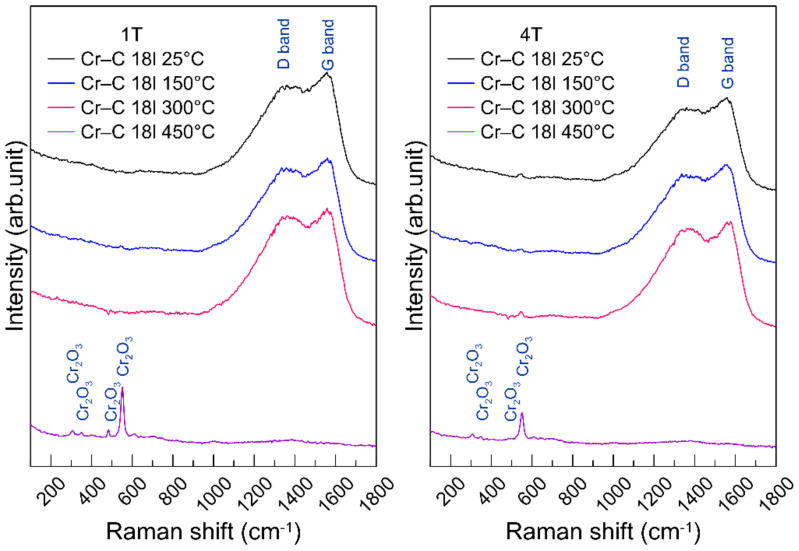
Raman spectra of the non-abraded regions of the CrC multilayer coatings after wear tests at 25 °C, 150 °C, 300 °C and 450 °C.

To investigate the thermal failure of the coatings, Raman spectroscopy was used to inspect the bondings of a-C:H top coat, and the results are shown in Figure 5. The Raman spectra of samples 1T and 4T at 25 °C, 150 °C and 300 °C show no obvious difference. The shift of the D band and G band is not detectable. However, at 450 °C, there are chromium oxides formed on the coating surface. The D band and G band of the carbon phase disappeared. This means that the a-C:H top coat was oxidized and completely decomposed. Although the coated sample lost 20~30% of hardness, the oxide on the surface provides a relatively lower friction coefficient at 300 °C and 450 °C. 

**Figure 6:**
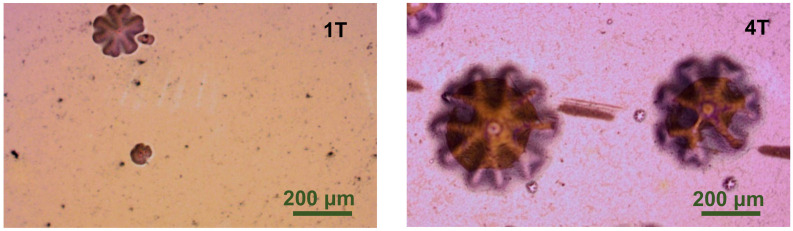
Optical microscope surface images of the non-abraded regions of Cr–C multilayer coatings of 1 quad-layer (**1T**) and 4 quad-layer (**4T**) deposited on the AISI M35 substrate after the 450 °C wear test.

Since the phase variation of a-C:H top coat was not detected on the coatings after being heated at 150 °C and at 300 °C, the damage could be initiated inside the periodic quad-layers or at the layer boundary. On the coated samples tested at 450 °C, there are some bumps with a 5-fold or 6-fold radial symmetry shape (Figure 6). It is not certain that the bumps are induced by the accumulation of gas due to the phase decomposition, the volume change induced by phase transformation, or the growth of oxide at the boundary. 

**Figure 7:**
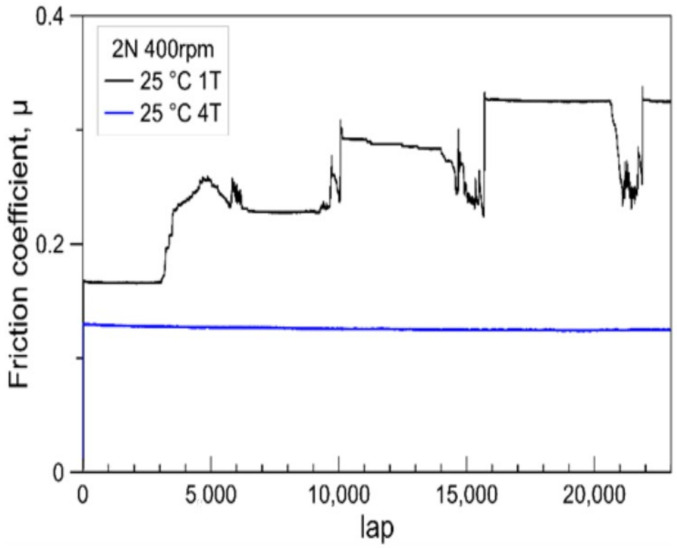
Friction coefficients depending on the lap number (cycle) for the tribological contact of an alumina ball (6 mm) in ball-on-disk testing with two different multilayer-coated AISI M35 under 2 N load at 25 °C.

In order to clearly identify the difference between the room temperature wear resistance of 1T and 4T coatings, the normal load of the ball-on-disc test was reduced to 2 N. Figure 7 shows the variation of friction coefficients in dependency of the sliding lap number for two coated samples under the 2 N load. The friction coefficient for the 1T sample increases stepwise. This reflects the penetration of each layer. The a-C:H top coat on 1T sample failed after 3000 laps. The whole multilayer coating of 1T sample was penetrated after 15,000 laps. On the contrary, the sample 4T endured 23,000 laps and remained a constant low friction coefficient. The average time-related depth wear rate [39] of samples 1T and 4T were approximately 41.8 nm/min and 7.1 nm/min. Such a big difference demonstrates the advantages of decrease the modulation period of the multilayer coating. Since the thicknesses of the a-C:H top layer of the two samples are the same, and the volume fractions of each material in the whole coating are also the same, the failure of coatings could be attributed to the crack propagation in the inner layers due to the stress. The decrease in the modulation period (more and thinner layers) causes more interlayer boundaries, which can effectively change the transverse cracks into longitudinal cracks and restrain the brittle failure of the coating [31,35,40]. This mechanism might be the reason for the superior wear resistance of the 4T coating sample. 

## 4. Conclusions

Carbon–chromium carbide–chromium tri-phase multilayer coatings with a difference modulation period were deposited by utilizing reactive high-power impulse magnetron sputtering, sequentially alternating the mixture ratios of ethyne and argon gas within the same constant deposition total pressure. The target pulse frequency, pulse duty cycle, average chromium target power, and total deposition time are all the same. Two different gas-mixture alternating periods were applied to obtain films with different numbers of layers and different layer thicknesses (modulation periods). After comparing the mechanical properties, the adhesion strength and the ball-on-disc wear behaviors of the multilayer-coated AISI M35 sample, some conclusions were drawn:

- Decreasing the modulation period can effectively adjust the elastic modulus and the subsequent ratio of hardness to elastic modulus (H/E) of the whole coating, which helps adapt the elastic strain in the coating. This improves the adhesion of the coating;
- The increased H/E value and layer boundaries for a smaller modulation period also restrain the cracking failure in the inner layers and provide a better wear resistance of coatings at room temperature.
- Due to the more severe decomposition of the hydrocarbon phase and oxidation of the chromium content in films, the wear-resistant performance of both coatings decreased with the increasing temperature. The difference in wear behaviors between the coatings of different modulation periods becomes inconspicuous at higher wear temperature.

## Data Availability

Data is contained within the article.
